# Development of a communication platform for patients with head and neck cancer for effective information delivery and improvement of doctor–patient relationship: application of treatment journey-based service blueprint

**DOI:** 10.1186/s12911-024-02477-4

**Published:** 2024-03-20

**Authors:** Yoo-Ri Koo, Eun-Jeong Kim, Inn-Chul Nam

**Affiliations:** 1 Department of Service Design, Graduate School of Industrial Arts, Hongik University, Seoul, 04066 Korea; 2 Department of Industry-Academic Cooperation Foundation, The Catholic University of Korea, Seoul, 06591 Korea; 3 Department of Otorhinolaryngology-Head and Neck Surgery, Incheon St. Mary’s Hospital, The Catholic University of Korea, Incheon, 21431 Korea

**Keywords:** Health communication, Health information systems, Information services, Health information management, Head and neck cancer

## Abstract

**Background:**

Effective communication and information delivery enhance doctor–patient relationships, improves adherence to treatment, reduces work burden, and supports decision-making. The study developed a head and neck cancer (HNC) communication platform to support effective delivery of information about HNC treatment and improve the doctor-patient relationship.

**Methods:**

This study was structured in three main phases: 1) The requirement elicitation phase sought an understanding of the HNC treatment journey and service failure points (FPs) obtained through patient/medical staff interviews and observations, along with a review of the electronic health record system; 2) The development phase involved core needs analysis, solutions development through a co-creation workshop, and validation of the solutions through focus groups; and 3) the proposed HNC communication platform was integrated with the current treatment system, and the flow and mechanism of the interacting services were structured using a service blueprint (SB).

**Results:**

Twenty-two service FPs identified through interviews and observations were consolidated into four core needs, and solutions were proposed to address each need: an HNC treatment journey map, cancer survivor stories, operation consent redesign with surgical illustrations, and a non-verbal communication toolkit. The communication platform was designed through the SB in terms of the stage at which the solution was applied and the actions and interactions of the service providers.

**Conclusions:**

The developed platform has practical significance, reflecting a tangible service improvement for both patients and medical staff, making it applicable in hospital settings.

## Background

### The growth of information and communication technology and the need for digital platform development

The use of information and communication technology (ICT) helps reduce medical costs, improve health outcomes, and increase patient satisfaction [1–3]. Information technology (IT) provides value-based healthcare and a seamless patient experience (PE) [4]. Health IT can be used effectively to address problems that may arise during the data collection process and integration with electronic health records (EHRs) or separate IT systems [5]. ICT is changing healthcare methods, targets, and content, such as the type of provision process, patient–provider interactions, and information management [6, 7].

Technology has transformed healthcare systems from hospital-to patient-centric, and the rapid growth of ICT has improved access to healthcare services [8]. In particular, ICT has had a positive impact on patients’ access to healthcare services and authorization, improving quality of life, empowering individuals to adhere to healthier lifestyles, and supporting social participation [9]. Therefore, ICT innovation plays a crucial role in increasing patient satisfaction with accessibility to healthcare services. Information systems and analytics research can also significantly contribute to the development of new and innovative health strategies and solutions [7, 10].

As of 2021, ICT development to treat cancer patients includes over 350,000 mobile health applications (apps) [11], and this field is growing every year [12]. However, despite the rapid growth rate of ICT, most data used for cancer treatment are not systematically organized in electronic systems [4].

Patient medical data are typically difficult to integrate, standardize, and use, as they often remain siloed in healthcare systems. Developing an integrated and innovative digital platform is crucial for enabling efficient healthcare services because lack of data-sharing restricts clinicians’ ability to establish integrated treatment paths, track patient behavior, and support self-management plans [9].

Regarding the digital healthcare platform, Hermes et al. [13], through a systematic review, classified the newly emerging roles of the health industry into six categories: Information platform, Platform for remote & on-demand healthcare, Data collection technology, Market intermediaries, Data management and analysis for healthcare providers, and Investors and consultants. Among these, it was found that most of the digital healthcare platforms, which have recently been on the rise, are focused on remote and on-demand healthcare. The purpose of the communication platform we targeted is effective information delivery and building a positive doctor-patient relationship, which is based on the active interaction between the stakeholders. Thus, the communication platform proposed in our study plays the role of an information platform.

According to the research results of Hermes et al. [13], the market share of information platforms is very low compared to other fields. Considering that seamless interaction between doctor and patient based on information delivery and emotional support is very important to improve the quality of care of HNC patients, the development of an information platform is of great significance. In addition, the digital platform has many functions such as information sharing, remote consulting and self-care app, and patient data management and analysis that must be considered integrated rather than presented independently for successful implementation in an actual healthcare setting.

Meanwhile, Parker et al. [14] and Jacobides et al. [15] mentioned that an advantage of a digital platform is the enhanced ability to promote interaction between communities comprising various stakeholders, including service users and service providers. However, due to the complexity of health information, access by third parties, including patients, is very limited, and this limitation prevents seamless interaction and communication between stakeholders due to knowledge barriers. In addition, due to a mechanism that fails to consider the perspectives of the medical staff who must actually operate the system, the platform developed with great cost and effort is often being ignored by the medical staff. Due to limited patient access to the system or non-application of the user-friendly interface, the platform has the problem of not functioning properly in actual hospital settings. Therefore, the development of an information platform that allows close interaction and seamless information delivery between patients and medical staff is necessary for the balanced development of the emerging digital healthcare industry.

### The digital healthcare platform for communication improvement

The recent studies on digital platforms to improve communication and interaction between patients and doctors include a mobile app to support patient self-management [16], a real-time decision tool and communication tool development [17], and implementation of a web-based telemonitoring strategy [18]. Thies et al. [16] identified barriers that arise related to the use of mHealth apps to improve clinical outcomes. As points of improvement, they identified that medical staff did not have enough time to check the app, patients did not know how to use it properly, and the app was not integrated with the existing EHR system. Patel et al. [17] developed real-time decision support tool integrated with EHR, communication tool between patients and medical staff, automated audit tool for feedback, and web portal to provide performance trends as interventions for quality improvement for risk management. Through the case study, they identified that, to increase the effectiveness of this intervention, it should be accompanied by activation of all team members and strengthening of IT infrastructure. Dijkstra et al. [18] explored the implementation strategy of web-based telemonitoring for teenagers. They conducted an evaluation based on seven domains to explore the applicability of the telemonitoring strategy they developed to hospital sites. As a result, face-to-face consultation between patients and doctors, simple data collection and management were identified as positive factors. Summarizing the above research results, it can be seen that various formats of digital platforms such as apps, communication tools, and telemonitoring systems are being developed to increase patients' satisfaction and quality of medical services. For their practical implementation, it is critical that simple data collection and management, active user participation, integration and interoperability with existing medical systems, and maintaining face-to-face contact service as important parameters.

In addition to the forementioned cases, the digital platform presented ways to improve communication between patients and medical staff in various forms: face-to-face coaching [19], home-based training program [20], web-based software for communication [21], telemonitoring [22], E-consult [23], Web-based lesson [24], and self-monitoring program [25]. However, most studies focused on telemedicine and often did not consider interoperability with existing EHR systems. In addition, the scope of operation of the platform was mostly limited on the premise of online interaction, making it challengeable to meet the practical needs of patients who prefer face-to-face contact.

### The importance of patient-medical staff communication in quality care

Enhancing communication between medical staff and patients is crucial for establishing optimal pain treatment and supporting high-quality pain self-management [26].

Head and neck cancer (HNC) requires a long treatment period and is associated with functional deficits in vocalization, speech, swallowing, and breathing. Older adult patients sometimes have a poor understanding of the disease and treatment process and may be unable to communicate verbally because of tracheostomy or other treatments. Medical staff have limited communication with patients owing to excessive workloads [27]. Effective patient communication is at the core of patient-centered treatment because understanding the treatment and interaction affects the bond between patients and doctors, increases the patient’s willingness to receive treatment, and leads to favorable treatment results [28]. Various stakeholders are involved in the treatment process for HNC, and vast amounts of complex information related to treatment are delivered to patients during the treatment journey. Effective information-oriented communication improves patient experience (PE) with HNC and health outcomes. Regarding the diagnosis system for Nasopharyngeal disease, as a type of HNC, Mohammed et al. [29] reviewed the existing diagnosis system and found that there was a need to explain the critical evidence of the existing systems, expand the required information in NPC diagnosis, and develop an interoperable diagnosis system. Kouroubali et al. [9] emphasized the necessity of seamless information sharing among all stakeholders in developing an integrated healthcare delivery platform for older patients. Considering that most patients with HNC are older adults, the development of an integrated healthcare platform is crucial. Such a platform should reflect the perspectives of both service users and providers, allowing efficient information sharing among stakeholders and comprehensive management of the data related to emotions or behavior. Communication should be enhanced by presenting a platform in which both perspectives contribute to seamless integration. Service blueprints (SBs) are often used as an effective methodology to support stakeholder collaboration. They map service processes and interactions between stakeholders from the user’s perspective [30–35] by visualizing a service system. This is beneficial for educating service users, evaluating service systems from various perspectives, and establishing specific response strategies for new service concepts or systems [36, 37]. The format helps service providers understand tasks and responsibilities and make effective decisions [35, 38, 39].

In the field of healthcare, there is a growing trend toward utilizing SBs to understand the service delivery process [40]. Healthcare is a complex domain with a large amount of medical information delivered to patients and requires seamless communication between patients and medical staff. This necessitates structural visualization of the quality and quantity of information provided to patients and the interaction between medical staff and patients. Visualizing the service delivery process can enhance stakeholder understanding and contribute to increased implementation effectiveness in the field.

### Study aim

This study aims to 1) develop an innovative user-centered communication platform that focuses on effective information delivery and improvement of the doctor–patient relationship aligned with the patients’ treatment journey with HNC, and 2) present a tangible mechanism through an SB on how the proposed platform can be applied to the current service system to improve HNC treatment.

The proposed platform utilizes SB to visually explain the service delivery process considering its implementation in the field and interoperability with current systems. Patient-centered communication platforms can increase patients’ willingness to receive treatment, improve treatment adherence, and induce positive treatment results by efficiently allocating doctors’ time and resources.

## Materials and methods

### Service blueprints as a practical method for service innovation

According to Bitner et al. [41], the use of SBs is a customer-centric approach to service innovation and improvement. They effectively visualize service processes, points of customer contact, and physical evidence related to services from the customer’s perspective to reveal potential points of improvement. As the global need for service innovation increases, developing and applying innovative tools and techniques to enhance customer experience in healthcare has become crucial. In a complex healthcare environment, improving PE requires not only the enhancement of the entire medical service process but also the refinement of individual stages.

Improving service processes requires a comprehensive understanding of all functional and psychological elements patients experience throughout their service journey. Key components included in building an SB are customer actions, on-stage (visible) contact employee actions, back-stage (invisible) contact employee actions, support processes, and physical evidence.

This study built an SB based on these five components to visually represent the service processes and systems of the proposed communication platform. The existing HNC treatment service processes and systems requiring service improvement before the introduction of the proposed platform were identified through the “As-is” SB, highlighting the service FPs. The improved service process including the proposed platform and operability with current systems were visualized using the “To-be” SB.

### Methods

**Fig. 1:**
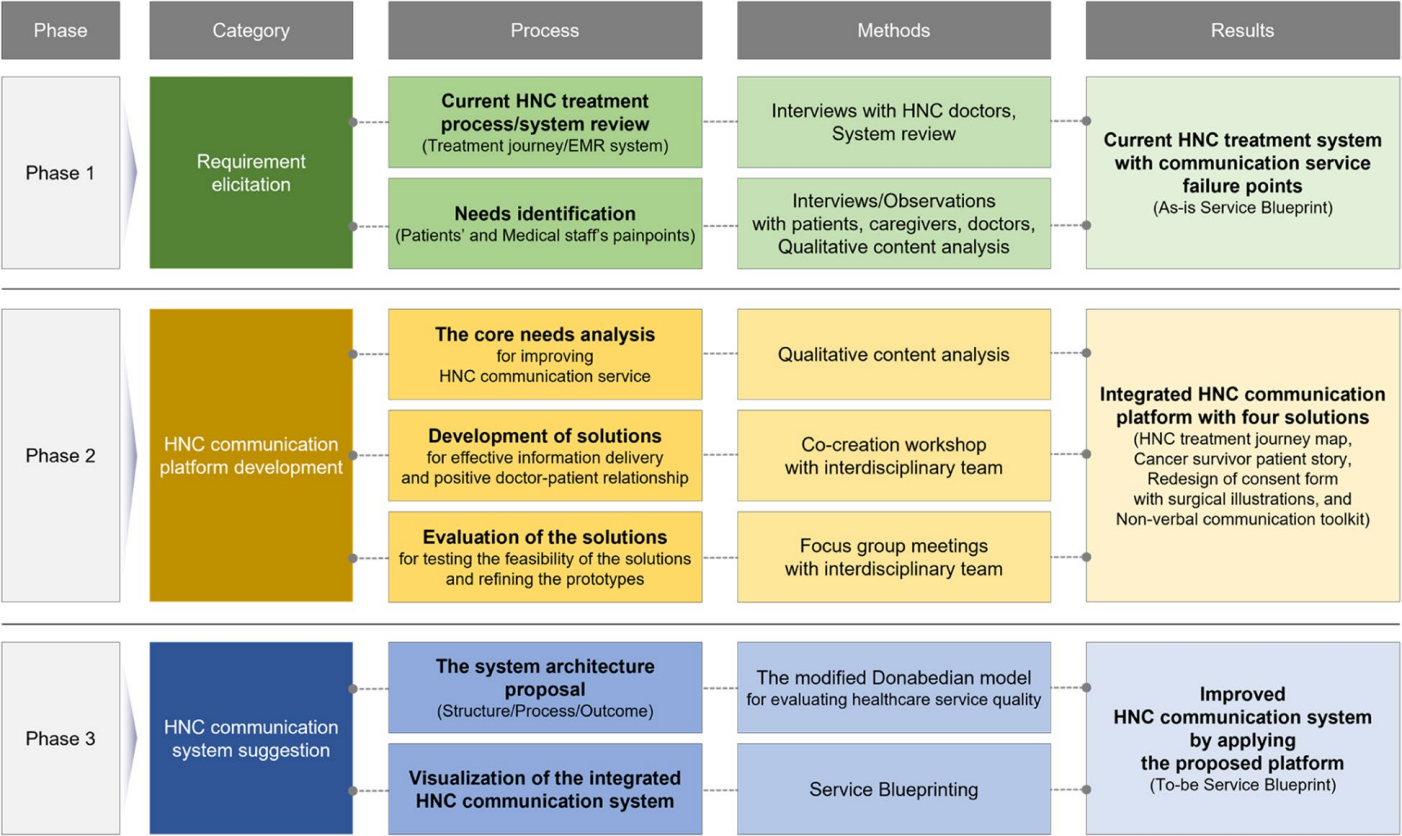
Diagram of the research flow

The study was conducted in three phases: 1) requirement elicitation, 2) HNC communication platform development, and 3) visualization of the improved HNC treatment process/system using the proposed platform (Fig. 1).

For the requirement elicitation phase (phase 1), the needs of patients and medical staff were identified, and the current HNC treatment process and EHR system were reviewed. Based on the identified needs and review, an As-is SB was created.

In Phase 2, the core needs for service improvement were derived from the service FPs. Then, a co-creation workshop was conducted with an interdisciplinary team to develop solutions corresponding to the core needs, and two focus group meetings were conducted with the HNC medical staff to evaluate the feasibility of the solutions. Finally, the four proposed solutions were integrated into an HNC communication platform.

In Phase 3, to specify and practice the mechanism such that the proposed platform could interoperate with the current hospital service system, the system architecture was created based on an adjusted model Donabedian framework of Structure-Process-Outcome (SPO) [42]. By integrating the proposed architecture and the As-is SB, the To-be SB presented how the current hospital service system could interoperate with the proposed platform and how the service FPs of the current service delivery process could be improved through the developed solutions.

In the Discussion section, we address which challenges should be solved for the suggested service system to operate seamlessly in an actual HNC treatment setting.

#### Phase 1: Requirement elicitation

##### Understanding the HNC treatment journey and current hospital system

First, we examined the HNC treatment journey and identified the structure and flow of the hospital's current EHR system. For patient actions, a five-stage treatment journey was identified through interviews with three HNC specialists. For physical evidence, service touchpoints at each journey stage were identified through observation. Additionally, the place, activity, patient's general psychology, workload of the medical staff, and doctor-patient interactions were identified at each stage.

The Catholic University of Korea Hospital was selected as a case study for reviewing the current HNC treatment process and EHR system. This university general hospital has a network of seven hospitals affiliated with the Medical Center and 35 departments, including Otorhinolaryngology, and 21 specialization centers. We selected this hospital considering the fact that many HNC treatments and surgeries are performed, making it easy to contact and recruit patients and medical staff for user research. In addition, the hospital is currently operating an EHR system by applying ICT, and as a large-scale hospital, it is making active efforts to improve patient-centered medical services, so it was judged to be a good fit for the topic of this study. By examining the EHR system provided by the hospital, we identified the processing and patient data management mechanisms for decision-making between departments at each stage. After reviewing the journey process and the hospital’s administrative system, an initial framework of the current healthcare system for patients with HNC was created.

##### Identifying healthcare service FPs

To identify patients' and doctors' pain points along the HNC treatment journey, we conducted nine in-depth interviews with 17 participants (five male patients, one female patient, five female caregivers, two female residents, and four male specialists) and five observations with 14 participants (four male patients, one female patient, two male caregivers, two female caregivers, four male specialists, and one female resident) between August and October 2021 (the complete data of the interviews and observations can be found in our previously published research paper [43]). For participant recruitment, a doctor who participated in one of the earlier interviews recruited interview participants from among patients and their caregivers who visited the outpatient clinic. Patients were conveniently recruited from patients who were about to undergo HNC surgery, who were hospitalized after surgery, or who were receiving rehabilitation treatment after surgery. Caregivers were recruited from among those who visited with the patient, and if the patient gave permission, additional participants were recruited from the patient's family. Informed consent was obtained from all the participants before conducting the interviews. The researchers collected data on patients’ specific care experiences based on their treatment journey and audio-recorded the one-hour in-depth interviews with participants’ consent. During the observations, we observed the doctors’ and patients’ main activities, clinical room space, doctor–patient interactions focusing on information delivery and communication, and the tools being used for communication. We videotaped the observations with participants’ consent.

We included caregivers in the interviews because most patients with HNC were older adults, and thus tended to be passive in expressing their opinions. We only included caregivers who had close relationships with the patients and could fully represent their perspectives. During interviews after surgery, conversation was impossible for patients because of the tracheostomy. A caregiver who could closely interact with a patient reflected the patient’s situation and perspective. Patients and caregivers participated in the interviews together or, depending on the situation, the caregivers or patients participated individually.

The identification of service FPs and categorization of the core needs used the qualitative content analysis method introduced by Graneheim and Lundman [44]. This method identifies meaning units based on meaningful sentences from the interview text, derives codes as keywords, and presents a final theme by categorizing them based on similar attributes of the keywords. Based on this method, the interviews and observational data were fully transcribed. The transcript was converted into meaning units of one or two sentences containing the patients' or medical staff's negative experience. Keywords were analyzed from each unit, and keywords with similar semantic properties were grouped into four core needs. Each meaning unit was identified according to the corresponding stage of treatment and its service FPs. We completed the As-is SB by determining the locations of the identified service FPs in the service flow of the framework presented in the previous phase.

Building the SB structure entailed identifying the care service process based on the patient journey and isolating service FPs from the structured service process, based on the framework introduced by Shostack [36, 37]. Blueprint maps generally differentiate between customer and service provider areas. The service provider area comprises areas where direct interaction occurs, and internal interactions occur behind the scenes. The flow between them is mapped by the lines of interaction, visibility, and internal interaction, and the direction of the flow is indicated with arrows. We used a basic framework to structure the information data management system and care service processes for patients with HNC into blueprints.

#### Phase 2: HNC communication platform development

A co-creation workshop and two focus group meetings were conducted to develop solutions for patient and medical staff service FPs. The participants comprised an interdisciplinary team of one specialist, three residents, a service design professional, three service design researchers, and an academic healthcare industry researcher. A face-to-face workshop was held on May 25, 2022, for approximately four hours. The workshop consisted of three stages: 1) in the orientation stage, participants were introduced to the 22 service FPs and four core needs identified from user research and made aware of the purpose of the workshop; 2) in the ideation stage, “How might we” (HMW) questions were introduced as a goal to meet the core needs, and participants were divided into two teams to brainstorm various ideas to solve the problem. Participants freely expressed their ideas by writing notes or drawing directly on the two printed A0-sized treatment journey maps (we simplified the map from the As-is SB for better understanding of the participants) posted on the wall using a pen and Post-it; 3) in the consensus and solution selection stage, participants reached a consensus of opinion by team at first, and all participants shared each team's opinion and reached a consensus among the teams to select the final four solutions.

The first focus group meeting (June 15, 2022) was conducted with HNC medical staff to evaluate the feasibility of the four selected solutions. One otolaryngology professor, two residents, and one outpatient and one ward nurse who did not participate in the previous workshop or focus group meeting were involved in this group meeting. They were comprised of people who were currently directly involved in HNC treatment and had experience participating in the hospital's program for PE and quality improvement (QI).In the first half of the meeting, participants were given an explanation of the four solutions selected in the co-creation workshop, along with the purpose, method, process, and content of the research. Afterwards, the participants evaluated each solution based on the ‘degree of goal achievement’, ‘degree of problem solving’, and ‘degree of meeting patient/medical staff needs’ of the developed solution, ‘logic of process and development’, and ‘appropriateness of methodology’ in the development of solutions, ‘ease of understanding’, ‘easy accessibility/usability’, and ‘flexibility of applications’ for utilizing the solution. These eight dimensions were drawn from the Donabedian model [45]’s seven dimensions for healthcare quality evaluation through the discussion among the co-creation participants to suit our study aim. At this stage, considering that the solution was in the early development stage, opinions were freely expressed on overall evaluation and feedback, related suggestions, and issues that may arise in the field, referring to the evaluation criteria rather than specific evaluation of each item. They evaluated 1) whether each solution solved the current service FPs, 2) whether the solution could be applied and implemented in the field, and 3) whether it was a suitable solution for the needs of older adult patients.

Subsequently, until the second group meeting, the participants who got involved in the co-creation workshop continued to materialize ideas by splitting the participants into small groups. While developing ideas, groups frequently exchanged opinions, and the entire team gathered and discussed information as needed. At the second focus group meeting (November 16, 2022, three hours), the specific content, format, and process of the four solutions developed thus far were reviewed. The participants of the second focus group were composed of the same as the first meeting. They were guided through the design brief of the specifically developed solutions and presented detailed opinions on each solution based on the eight evaluation criteria used at the first meeting. The three participants freely suggested individual opinions and then shared their opinions on each solution to reach a consensus. During the focus group, they suggested recommendations on each criteria that needed improvement or supplementation. They discussed the priorities that should be considered when each solution is implemented in the actual healthcare setting. Finally, we proposed four experience prototyping methods as final solutions to communicate information effectively and improve doctor–patient relationships. The solutions were presented as an HNC communication platform in conjunction with the hospital's current system.

#### Phase 3: visualization of the improved HNC treatment process/system by applying the proposed platform

To ensure the proposed platform could interoperate with the current hospital service system, a system architecture was created based on the adjusted model of the Donabedian framework of SPO. This model comprises three components for evaluating the quality of healthcare: Structure as requirements, Process as actions, and Outcomes as end results. Tossaint-Schoenmakers et al. [42] improved and presented criteria for the three components to be applicable to the recent digital healthcare service environment with the emergence of e-health, as shown in Table 1.

**Table 1:** The adjusted Donabedian model for E-health service evaluation

Components	Structure	Process	Outcome
Criteria	-resources -human resources -organizational structure	-technical actions -interpersonal actions -management of the actions	-health status -satisfaction -efficiency

Based on this framework, we proposed system architecture for improving HNC treatment services. The four proposed solutions for the HNC communication platform and the core needs for service improvement, encompassing the needs of both patients and medical staff, are combined and presented in the three categories of Structure, Process, and Outcomes.

By integrating the proposed architecture and the As-is SB, the To-be SB presented how the current hospital service system could interoperate with the proposed platform and how the service FPs of the current service delivery process could be improved through the developed solutions.

The four solutions were positioned at their respective journey stages, and the interactions between the medical staff and patients at each stage were explained based on their actions. Subsequently, the flow and interactions of visible or invisible contact employees with patients in terms of the four solutions were visually represented using arrows and icons. The symbols indicate how the proposed solutions address the service FPs identified in the As-is SB aimed at service improvement.

## Results

### Phase 1: requirement elicitation

#### Current HNC treatment process system review

Based on the interviews with three otolaryngologists, the treatment journey for HNC was divided into five stages. The first stage, “Cancer diagnosis and staging work-up,” occurs when patients visit a hospital and are diagnosed with cancer after conducting staging studies. The second stage, “Preoperative counseling,” is a decision-making stage in which the test results are reviewed, optimal treatment methods are determined and explained to the patient, and the treatment direction is decided together with the patient. The third stage, “Obtaining informed consent,” is when the extent of the surgical treatment, the methods, and possible complications are explained to the patient before surgery. The fourth stage, “Surgery and recovery,” correspond to the process of recovery after surgery, including treatment in the ICU and general ward. The fifth stage is “Rehabilitation and follow-up,” in which surveillance for disease recurrence is performed, and rehabilitation treatment is given through periodic outpatient attendance after discharge.

Based on the EHR system investigation at the hospital, patient information for registration, referrals to other departments for operability check-ups, and data on evaluation results were processed in the first stage, and ordering and conducting diagnostic evaluation tests were made based on these data. In the second stage, information on diagnoses, treatment methods, and surgery schedules were shared between medical staff and patients. In the third stage, the surgical process and risks were explained, and informed consent was obtained from the patient. In stage four, hospitalization for surgery, postoperative evaluation, and support departments’ consulting data were processed. Based on this information, interactions such as prescribing injections and medication for pain control, disinfecting surgical wounds, responding to patient needs, and explaining postoperative conditions were made. In stage five, medical assessment results, postoperative evaluation data, and medical appointments for outpatient clinic-related data were processed. This involved guiding the patients through rehabilitation therapy, conducting follow-up evaluations, and explaining their postoperative conditions. The service delivery process of the EHR system and contact employee actions according to the HNC treatment journey are summarized in Table 2.

**Table 2:** Service delivery process, contact employee actions, and service FPs on the HNC treatment journey

**HNC treatment Journey stage**	**Service delivery process**	**Contact employee actions** (P: physician, R: resident, N: nurse)	**Service FP** **(Patients’ perspective)** (F: patient service FP)	**Service FP** **(Medical staff’s perspective)** (F’: medical staff service FP)
Stage 1 (Cancer diagnosis and staging work-up)	-Registering patient information	(P) Suspecting cancer, ordering diagnostic evaluation tests (R) Symptom/visit record review/report (N) Confirm Reservation /care guide	(F1) Lack of adequate explanation of the need for cancer diagnosis tests and the treatment procedures	(F’1) The burden of repeatedly explaining the diagnostic procedures to patients to persuade them get tested
	-Referral to other departments	(P/R) Reviewing medical records (N) Reviewing evaluation test order	-	-
	-Reporting initial medical assessment results	(R) Running diagnostic evaluation tests (N) Confirming reservation information on departments to visit (P/R) Reviewing medical records	(F1) Lack of adequate explanation of the need for tests for cancer diagnosis and the treatment procedures	-
Stage 2 (Preoperative counseling)	-Reporting diagnosis and treatment methods	(P) Cancer diagnosis (P) Explain treatment process and methods (N) Confirming Reservation /care process guide (P/R) Review medical records	(F2) limited Access to the Internet for comprehensive, reliable HNC information (F3) Lack of comprehensive explanations of the entire treatment process (F4) Severe shock after being diagnosed with cancer, anxiety about surgery, hard to find patient survivor stories	(F’2) Sudden cancellation of surgery or delays in decision-making due to distrust of the doctor’s suggestions (F’3) Burden of repeatedly explaining treatment methods to older patients until they fully understand
	-Reporting surgery schedule	(P) Scheduling for surgery (N) Confirming/scheduling Reservation (P/R) Reviewing medical records	-	-
Stage 3 (Obtaining informed consent)	-Reporting informed consent	(P) Explaining surgical process and risks (R) Printing out consent forms and explaining the meaning of informed consent (P/R) Reviewing medical records	(F5) Lack of information about post-surgery care plan, rehabilitation, and post-discharge management	-
	-Managing consent form (review/print)	(R) Explaining informed consent (P/R) Reviewing medical records	(F6) Having anxieties and worries about unpredictable surgery results after hearing the explanation of the long and complicated contents of the consent form (F7) Text too small on the consent form, and too much content, making it difficult to understand the main point and focus on important content (F8) Content that is difficult to understand through text alone, complex and long explanations that are difficult to understand after hearing them once	(F’4) The burden of explaining long and complex consent forms to older patients and helping them understand the contents
	-Managing consent form (review/scan)	(R) Scanning the signed forms (N) Confirming/scheduling Reservation (P/R) Reviewing medical records (N) Checking patient admission and surgery schedule	-	-
Stage 4 (Surgery and recovery)	-Reporting surgery hospitalization data	(P/R/N) Performing surgery	-	-
	- Reviewing and reporting on postoperative evaluation	(R) Prescribing medication for pain control (N) Responding to patient needs, administering pain control, disinfecting surgical wounds (P/R/N) Reviewing medical records	(F10) High level of anxiety about the results of surgery and recovery due to pain and difficulty speaking, Difficulty finding other patients’ successful rehabilitation stories (F11) Difficulties in vocalization and limited communication due to tracheotomy, resulting in passive expression of needs (F12) Difficulty communicating with pen and paper, emotional fatigue after surgery (F13) Passive and simple communication using gestures due to vocalization difficulties	(F’5) Difficulty quickly identifying and responding to the pain and condition of patients who do not actively express it
	-Consulting support departments	(P) Explaining postoperative conditions, ordering postoperative evaluation tests (R) Update on postoperative condition (N) Discharge information guide (P/R) Reviewing medical records	(F9) Slow update on postoperative progress, and no information about the changes in symptoms during recovery	(F’7) Lack of opportunities to provide emotional support and motivation to prevent patients from giving up treatment
Stage 5 (Rehabilitation and follow-up)	-Reporting medical assessment results	(R) Guide to rehabilitation therapy	(F15) Increased incidence of depression due to the burden of self-managing long-term rehabilitation and a need for social/psychological support from people around the patients	(F’6) Contact with patients only in outpatient clinics, difficulty in providing additional rehabilitation information
	- Reviewing and reporting on postoperative evaluation	(P) Ordering follow-up evaluation tests, explaining the postoperative condition (N) Scheduling/guide on the evaluation tests (P/R) Reviewing medical records	(F14) Limited access to information on cancer metastasis, treatment progress, rehabilitation treatment, and self-management after discharge	-
	-Managing medical appointments	(N) Scheduling/managing notifications	-	-

#### Needs identification

In Stage 1 (Cancer diagnosis and staging workup), two service FPs were identified. The patient service FP appeared as lack of adequate explanation of the procedures needed for cancer diagnosis (F1) in relation to the process of diagnostic evaluation tests. The service FP for medical staff was found to be the burden of repeatedly explaining the diagnostic procedures to patients to persuade them to get tested (F’1). In Stage 2 (Preoperative counseling), three patient service FPs and two medical staff service FPs were identified. Patients reported difficulties searching for information on cancer treatment on the Internet after a cancer diagnosis (F2), lack of explanation of the entire treatment process (F3), and anxiety (F4) as important service improvement points, which were the following aspects: information search, information content, and negative emotions. The medical staff viewed distrust between the patient and the medical staff as causing sudden surgery schedule cancellations or delays in decision-making (F’2), and patients’ low level of understanding (F’3) as service FPs.

In Stage 3 (Obtaining informed consent), four patient service FPs and one medical staff service FP were identified. Patients saw lack of proactive information provision about the post-surgery care plan and rehabilitation (F5), anxiety about surgery (F6), small text that was hard to read (F7), and text-based information delivery that reduced patient understanding (F8) as their critical service FPs. Medical staff considered the difficulty in helping patients understand complex information (F’4) a core service FP.

In Stage 4 (Surgery and recovery), there were five service FPs: slow information updates and insufficient information on symptom changes (F9), high levels of anxiety after surgery (F10), difficulties in vocalization and passive needs expression (F11), and difficulties in verbal communication and no alternative means to communicate (F12, F13). The medical staff identified two service FPs: difficulty in giving quick responses to patients’ pain and needs when verbal communication is limited after surgery (F’5), and lack of opportunity to provide emotional support and motivation for treatment (F’7).

Stage 5 (Rehabilitation and follow-up) included two patient service FPs and one medical staff service FP. The patients mentioned limited access to comprehensive post-surgery information (F14) and the burden of long-term rehabilitation and self-management after discharge with no emotional support (F15) as critical service improvement points. The medical staff viewed the interruption of information provision after the patient was discharged (F’6) as a problem.

**Fig. 2:**
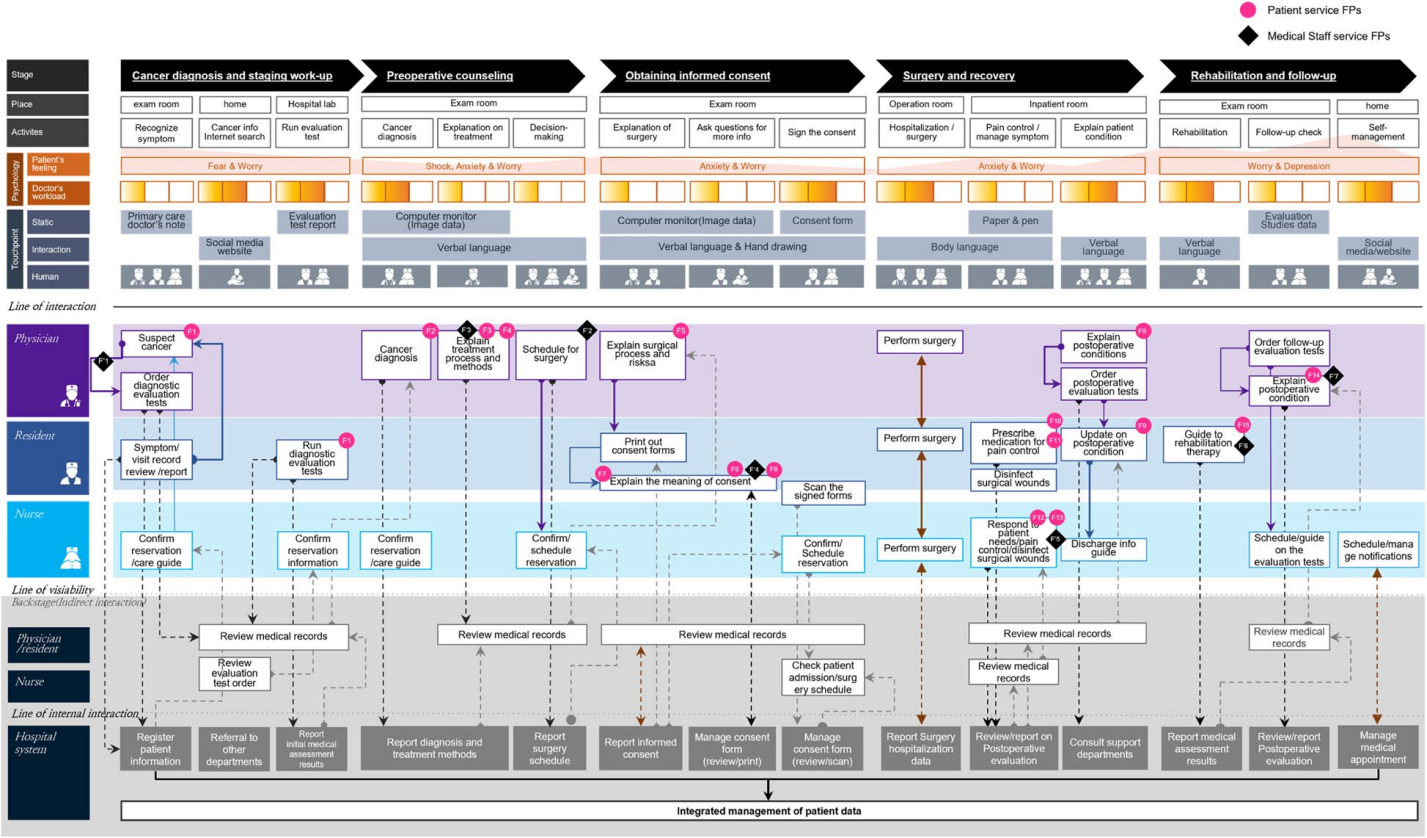
Current communication and information delivery system with healthcare service FPs (As-is SB)

Patient and medical staff service FPs identified by journey stage are presented in Table 2. Communication service FPs in the current HNC treatment system were compiled into an As-is SB (Fig. 2). Contact employee actions were divided into three tiers—visible, invisible, and support, according to SB principles, and the interactions between the parties were indicated by arrows. Patient service FPs were largely distributed in journey Stages 2–4, and there was a high need for service improvement on the aspect of information delivery, such as collecting more information, reliable information, proactive and comprehensive information, and non-verbal communication support. Throughout the journey, the medical staff highlighted the relationship-centered difficulties of explaining information and convincing patients to accept tests and treatment.

### Phase 2: HNC communication platform development

#### Core needs analysis

After grouping the service FPs based on the similarity of their attributes, four core needs were identified (Table 3). F1–3, F5, F9, and F14 from patients and F’1, F’2, and F’6 from medical staff mentioned the poor delivery of information at each stage of the treatment journey, such as information on the need for evaluation studies, difficulties in searching the Internet for comprehensive and reliable cancer information, explanations of treatment methods and processes, guidance on precautions before surgery, guidance on surgery and recovery processes, and postoperative observation and guidance. These service FPs emphasized the need to obtain information promptly. There was also a need for better information delivery to understand the overall flow of the journey, to plan, or make informed decisions. Moreover, a need for a method to access and check care information anytime and anywhere was required. These service FPs were linked to the content and quality of treatment information delivered to the patient and were synthesized into the need for *comprehensive information delivery and easy access to care information*.

**Table 3:** The core needs of patients and medical staff based on their service FPs

**Core needs**	**Journey stage**				
	**1 (Cancer diagnosis and staging work-up)**	**2 (Preoperative counseling)**	**3 (Obtaining informed consent)**	**4 (Surgery and recovery)**	**5 (Rehabilitation and follow-up)**
*Comprehensive information delivery and easy access to care information*	F1, F’1	F2, F3, F’2	F5	F9	F14, F’6
*Emotional support to relieve negative feelings*	-	F4	F6	F10	F15, F’7
*Information visualization to better understand the contents*	-	F’3	F7, F8, F’4	-	-
*Non-verbal communication tool to support active and prompt needs expression*	-	-	-	F11-F13, F’5	-

F: patient service FP, F’: medical staff service FP

F4, F6, F10, and F15 from patients and F’7 from medical staff corresponded to patients’ physical pain and negative psychological state at each stage of their treatment journey. Patients expressed anxiety about the unexpected cancer diagnoses, uncertainty about surgical results, postoperative pain, slow recovery, complications, and long-term rehabilitation. Patients with HNC had a great sense of age-related psychological decline, and it was reported that it was not easy to obtain psychological support throughout the long-term treatment process because they had few family members or close caregivers around them. Therefore, emotional support to relieve negative feelings is necessary to assist patients to actively engage in treatment without abandoning it. These service FPs are about processing and responding to patients’ negative emotions during treatment. They can be synthesized into a need called *emotional support to relieve negative feelings*.

F7 and F8 from patients and F’3 and F’4 from medical staff show that patients have difficulty understanding complicated and lengthy information through verbal explanations when signing consent for surgery. Additionally, it was difficult to remember what was heard and effectively share it with caregivers and family members. This suggests that in order for patients to quickly understand and remember the content information must be presented visually as well as orally and as text.

F11–F13 and F’5 show that patients are passive in expressing their needs during hospitalization, and their communication methods are limited. Consequently, there is a need for an efficient tool that can facilitate quick and active communication. Therefore, service FPs related to communication and interaction in a state where verbal communication was not possible after a tracheostomy were synthesized as a need for a *non-verbal communication tool to support the active and prompt expression* of needs.

#### Developing an HNC communication platform to provide solutions

Table 4 shows the process of development of solutions for effective communication and information delivery for patients with HNC. Focusing on the four core needs identified, the workshop participants discussed how each need could be met and how to address the service FPs. Using the HMW questions presented at the beginning of the workshop, the participants brainstormed and discussed various design ideas for solutions.

**Table 4:** Development process of the four solutions for effective information delivery and improvements in communicating with HNC patients’

**Core needs**	**Service FPs**	**HMW**	**Design ideas**	**Solutions**
Core Need 1. *Comprehensive information delivery and easy access to care information*	Absence of reliable information at each stage of the treatment journey (Stages 1–5)	How can patients conveniently access reliable, comprehensive HNC information anytime, anywhere?	-Provide information on diagnostic tests, treatment methods and process, and postoperative conditions at each stage of the treatment journey on the patient journey map -By identifying the patient’s emotional curve along the journey, encourage patients to prepare for the journey in advance -Connect scattered information on a website accessible through a QR code embedded in the journey map -Connect to external institutions other than hospitals that provide medical information	Solution 1. HNC treatment journey map
Core Need 2. *Emotional support to relieve negative feelings*	Fear of cancer and psychological anxiety after cancer diagnosis (Stages 2–5)	How can patients reduce their anxiety about death and the treatment process?	-Provide stories about patients after receiving a cancer diagnosis -Provide representative patient types for indirect experience -Connection with specialized institutions to overcome cancer patients’ anxiety	Solution 2. Cancer survivor stories
Core Need 3. *Information visualization to better understand the contents*	Need to improve delivery of information on HNC treatment methods that are difficult to understand (Stages 2–3)	How can patients easily understand explanations about treatments?	-Hierarchical arrangement and notation of surgical procedures to help patients understand -Utilize human body illustrations and models that are easy to understand -Redesign the consent form for surgery considering the characteristics of older adult patients	Solution 3. Redesign consent form and use visual Surgical illustrations
Core Need 4. *A non-verbal communication tool to support active and prompt needs expression*	Difficulties in direct communication due to discomfort and problems with pronunciation and vocalization after surgery (Stage 4)	How can patients quickly convey their opinions and needs to the medical staff without using verbal communication?	-Provide a communication toolkit using frequently used words and sentences for patients in hospital -Guidance on communication channels (inquiries, phone, fax, etc.) where patients can receive immediate feedback	Solution 4. Non-verbal communication toolkit

Regarding *comprehensive information delivery and easy access to care information* (Core Need 1), participants suggested providing information for each stage of the patient journey, considering the patient’s emotional state, involving direct information delivery through a QR code, and linking information from external organizations. The participants gathered opinions on the proposed ideas to produce a treatment journey map that could comprehensively cover this information at each journey stage.

Regarding *emotional support to relieve negative feelings* (Core Need 2), participants suggested collecting information on other patients with HNC and introducing a virtual PE to help new patients understand the treatment journey. Additionally, a method for guiding patients by connecting them with a professional psychological counseling center was suggested to overcome their anxiety. Regarding *information visualization to better understand the content* (Core Need 3), the participants suggested a simple list of information in the consent form to be classified in a hierarchy according to importance, the use of medical illustrations and models instead of drawings, larger fonts that are easy to read, and the design of the document emphasizing color contrast. Regarding *a non-verbal communication tool to support active and prompt needs expression* (Core Need 4), it was suggested that patients be provided with a list of questions and requirements for the most frequently addressed issues and complaints during hospitalization and to provide immediate feedback to patients. Methods of diversifying communication channels and giving patients the right to choose their options were also discussed.

The four solutions proposed by the co-creation workshop corresponded to the HNC treatment journey map, cancer survivors’ stories, operation consent redesign and surgical illustrations, and a non-verbal communication toolkit.

The HNC treatment journey map (Solution 1) consisted of six sections: introduction of a five-stage treatment journey, patient emotion curve, treatment information for each journey stage, precautions, a storyboard for emotional support, and contact information for related organizations and departments. The journey map included essential information that patients wanted or needed to know for each journey stage and allowed them to identify and understand all the information along their treatment journey.

For Cancer survivor stories (Solution 2), to help patients receive comfort from others’ stories, three typical types of HNC (laryngeal, oropharyngeal, and tongue cancers) were defined and developed into personas. For each persona, a 10-panel storyboard was created, focusing on the patient’s treatment experience and psychology.

For the redesign of the operation consent form and surgical illustrations (Solution 3), important information was highlighted and visual contrast was applied to critical sections using gray boxes. Thirteen surgical illustrations showing surgical sites and procedures commonly used in HNC treatment were presented simply and clearly.

The non-verbal communication toolkit (solution 4) took the form of an augmentative and alternative communication (AAC) system organized into five categories (greeting and answer, pain and symptoms, mood and feeling, question, and request). The AAC categories were developed by combining a literature review of the AAC system, analysis of items frequently requested by patients during hospitalization, and feedback from HNC specialists.

#### Evaluating the feasibility of the solutions

Two focus groups were conducted to evaluate the feasibility of the proposed solutions. At the first meeting, the interdisciplinary team discussed three aspects of the solutions: 1) whether each solution improved the identified service FPs and met core needs, 2) whether each solution was practical and applicable to actual hospital settings, and 3) whether each solution was designed considering older adults, who comprise the majority of HNC patients.

Participants agreed that the four proposed solutions improved the current service FPs and sufficiently met the core needs. Regarding field applicability, the participants suggested the information provided should be focused on content directly related to HNC treatment to prevent patients from being confused by excessive information. For a patient-friendly approach, participants were generally in favor of the solution being presented on a digital platform but emphasized that offline accessibility should be included in consideration of older patients who are less able to access digital technology. Therefore, it was suggested that some of the solutions, such as the maps and toolkits, should be provided not only as apps or on websites but also as printed versions, considering the patients’ ability and preferences for information access. It was also mentioned that structured visualization is necessary to improve the understanding of information provided mainly through text.

The four solutions were subsequently redeveloped based on the results of the discussions at the first meeting. For the HNC treatment journey map (Solution 1), as much unnecessary information as possible was removed, and the page was organized around information directly related to HNC treatment. The content was verified by an HNC specialist for accuracy of meaning, appropriateness of terminology, and the inclusion of key content. The patient’s emotions and the medical staff’s workload were added to the top of the map so that the emotional aspects of the patient and medical staff could be identified and empathized with at each journey stage.

For Cancer survivors’ stories (Solution 2), useful information such as the persona’s physical symptoms, emotional state, commonly experienced treatment processes, and frequently asked questions were added.

For the redesign of the operation consent form with surgical illustrations (Solution 3), surgical illustrations were developed by adding image zoom and memo functions so that doctors can use them as interactive visual explanatory material for patients.

Regarding the Non-verbal communication toolkit (Solution 4), to maintain overall unity across the communication platform, illustrations for each sentence were redesigned using the personas developed from cancer survivors’ stories, and a few sentences were added or modified through feedback from HNC medical staff.

In the second focus group, additional supplementary details for the four solutions were discussed, and brainstorming was conducted on the appropriate solution delivery methods and media, and how doctors could use the solutions for effective information delivery and positive doctor–patient relationships. These four solutions were provided as digital materials that could be freely accessed in the form of apps and websites. However, considering the digital inexperience of older patients, the journey map was also created as foldable leaflets, and the communication toolkit was designed as an easy-to-hold booklet. Cancer survivors’ stories were discussed to introduce the storyboard as a section on the journey map, with additional information being available through the QR code.

Finally, the four final solutions, completed by iteratively supplementing and developing the prototypes, were proposed as an integrative system model called the HNC communication platform.

**Fig. 3:**
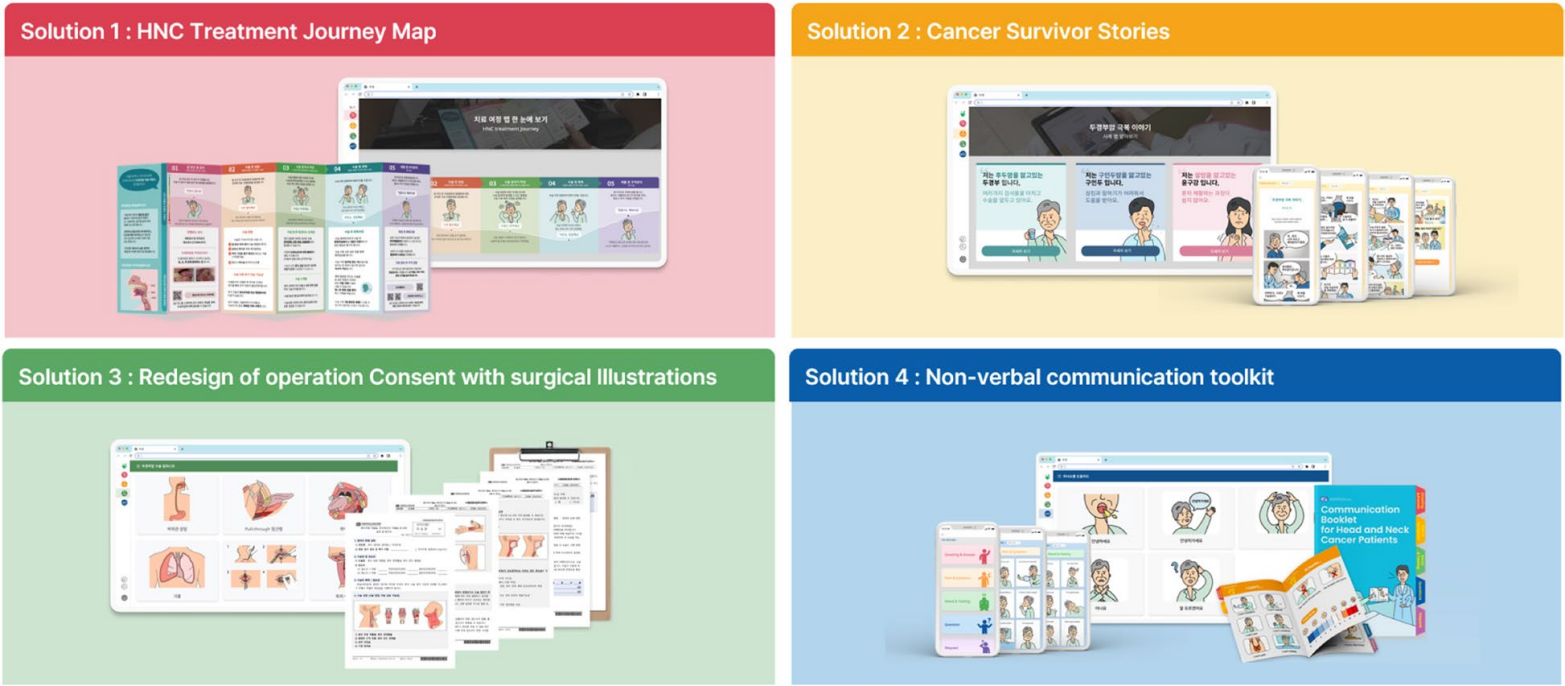
The four solutions developed for HNC communication

The communication platform comprising the solutions was submitted to two world-class design awards and won the Red Dot Design Award in 2022 and the iF Design Award in 2023, which demonstrates the effectiveness and value of the design as a prototype for improving healthcare PE^1^ (Fig. 3).

### Phase 3: developing an integrative HNC communication system

#### Building the system architecture

A system architecture was built to demonstrate how the proposed platform could be applied to and interoperated as an integrated communication system. The To-be SB was used to visually structure the integrative communication system, and Donabedian’s SPO framework was used to structure the components to create the SB.

The structure showed the critical requirements, including the information data categories and how information was presented and delivered for each solution. This process showed how the four solutions worked for each HNC treatment journey stage, focusing on contact employee actions. The outcomes included patient and medical staff satisfaction, improvements in medical services, and expectations of what could be achieved when the four solutions were embedded in the current treatment system (Table 5).

**Table 5:** A system architecture for HNC communication system

**Solution**	**HNC treatment journey map**	**Cancer survivor stories**	**Consent form redesign and surgical illustrations**	**Non-verbal communication toolkit**
**Goal**	*Comprehensive information delivery and easy access to care information*	*Emotional support to relieve negative feelings*	*Information visualization to better understand the content of information*	*A non-verbal communication tool to support prompt active expression of needs*
**Structure** *(Requirements)*	-The necessity for diagnostic tests -The content and flow of the entire treatment process -Precautions before surgery -Care plan after surgery -Management of rehabilitation after discharge	-Personas (representative patient types) -Patient’s typical physical symptoms and emotional states - Procedures at each stage of the treatment journey - Medical staff's explanation of the treatment process and methods - What patients need for emotional support -Cancer survivor storyboard for patients	- Different colors according to the hierarchy of information - Additional explanations for medical terminology in lay terms - Surgical illustrations to explain treatment methods and sites for surgery - Key points underlined and highlighted in bold - Improved readability using larger fonts and increased line spacing - Illustration of surgical procedures expressed simply	- Booklet or app - Patient’s requirements during hospitalization categorized - A combination of simple language, and images and symbols that reflect emotions - Material available in multiple languages
**Process** *(Actions)*	(Stage 1) Provide information about cancer and overview of treatment procedures (Stage 2) Introduce comprehensive information about the treatment journey (Stage 3) Give information on surgical methods, precautions, and care plans (Stage 4) Explain postoperative progress, pain management, and rehabilitation (Stage 5) Provide information on post-discharge self-care, and psychological and social support	(Stage 1) Introduce network for sharing personal feelings and empathy (Stage 2) Provide indirect experience of the treatment journey using personas (Stage 3) Evoke empathy from others by sharing personal anxiety about surgery (Stage 4) Share personal experience of pain, complications, and concerns about the recovery period (Stage 5) Share difficulties in self-management, rehabilitation, and survival stories for encouragement	(Stage 2-3) Use simplified visual images of surgical sites and procedures (Stage 3) Use of consent form with abbreviated key contents and important items emphasized	(Stage 4) Provision of non-verbal communication tool combining images and keywords
**Outcome** *(End results)*	- Acquisition of reliable and accurate information - Increased work efficiency and reduced workload for doctors due to reduction of repetitive explanations	- Reduction of patient’s anxiety - Strengthening of emotional support through empathy between people with similar experiences - Making a quick and positive decision for treatment based on trust between doctor and patient	- Improved patient understanding of treatment - A more positive attitude toward treatment from patients	- Patients willing to communicate quickly and actively - Rapid and appropriate response by doctors to patient needs - Positive interaction between patients and medical staff

#### Visualizing the HNC communication system integration on the proposed platform

The proposed HNC communication platform was integrated into the current service system by applying components of the system architecture to the As-is SB, showing the current HNC treatment system and service FPs. Figure 2 shows the To-be SB that explains the integrated communication system. It shows the service FPs where the proposed communication platform is applied to the current treatment system and explains how the solutions operate, interact with the current system, and improve service.

*HNC treatment journey map* (Solution 1) was applied to the entire treatment journey from Stages 1 to 5 and provides information that patients need at each stage. This was meant to provide comprehensive information delivery and easy access to care information (Core need 1).

At Stage 1, the map provided an understanding of the information about cancer and the overall treatment plan, enabling patients to understand the treatment process through diagnostic testing and determining treatment methods. Medical staff could effectively communicate treatment information to patients by using the map. In Stage 2, comprehensive treatment information was provided, allowing patients to understand the entire treatment plan in advance, and engage in decision-making along with their families. At Stage 3, information on surgical methods, precautions, and care plans was provided to help patients understand and prepare for the postoperative treatment process. In Stage 4, information on postoperative progress, pain management, and rehabilitation was offered, allowing patients to receive rapid updates on their surgical progress and plans for rehabilitation after discharge. At Stage 5, information on post-discharge self-care and psychological and social support was provided to enable patients to continue rehabilitation therapy and self-management at home connected to the hospital.

*Cancer survivor stories* (Solution 2) applied to Stages 2–5. For emotional support to relieve negative feelings (Core need 2), various methods for building empathy with patients were presented by stage. In Stage 1, before the patient fully engaged with the solution, the medical staff introduced an emotional support network, including cancer survivor stories, allowing the patient to access and explore the relevant information in advance. This provided a preliminary overview for the patient. In Stage 2, the three developed personas were employed to enable patients to indirectly experience the upcoming treatment process. These personas provided insights into possible treatment journeys that patients may encounter. In Stage 3, representative symptoms, pain, and psychological aspects were introduced. By presenting potential emotional changes and the survivors’ determination to overcome them through storytelling, patients could be encouraged to relate to the survivor stories of the personas, thereby reducing anxiety about surgery and fostering a more positive mindset. In Stage 4, the solution addressed typical characteristics of postoperative pain, complications and changes in symptoms following surgery. This solution also included questions frequently asked by patients along with responses from healthcare professionals, aiming to alleviate patient anxiety about their situation and establish a foundation of trust in the medical team. In Stage 5, the solution encouraged patients to persist in rehabilitation therapy and self-management after discharge by continuously updating and introducing new patient stories, fostering a commitment to maintain these practices. Through this solution, medical staff could directly or indirectly convey stories and information that can boost a patient’s positive mindset. This would ultimately create a treatment environment in which patients are more likely to adhere to medical decisions and treatment regimens. Additionally, the journey map allows patients to immediately submit feedback or complaints to the QI team through an embedded QR code, and medical staff and the QI team can quickly check and respond to the complaints. This facilitates timely interaction between patients and medical staff.

*Redesign of operation consent form and the use of surgical illustrations* (Solution 3) apply to Stages 2–3. To help patients better understand informational content, the form was redesigned to emphasize the key content through highlights, visual contrasts, increased font size, sentence rearrangements, and the addition of simple surgical illustrations. At Stage 2, healthcare professionals could effectively explain the proposed surgical site and treatment methods to patients using simplified surgical illustrations, thereby facilitating patient understanding. In Stage 3, through the redesigned operation consent form, patients could concentrate more effectively on essential information and gain a better understanding of the content through surgical illustrations. Healthcare professionals could communicate information efficiently to patients through structurally visualized content and illustrations.

The *non-verbal communication toolkit* (Solution 4) was applied at Stage 4. To support non-verbal communication and facilitate effective communication, a communication booklet based on the AAC system was developed. Using it, patients could convey their thoughts and requirements to medical staff quickly and accurately. The medical staff could then respond promptly by addressing their needs. To facilitate timely interaction between patients and medical staff, when a patient clicks on a specific sentence based on his or her needs, the medical staff immediately receives notification on the EHR system allowing them promptly address the needs of patients in response.

**Fig. 4:**
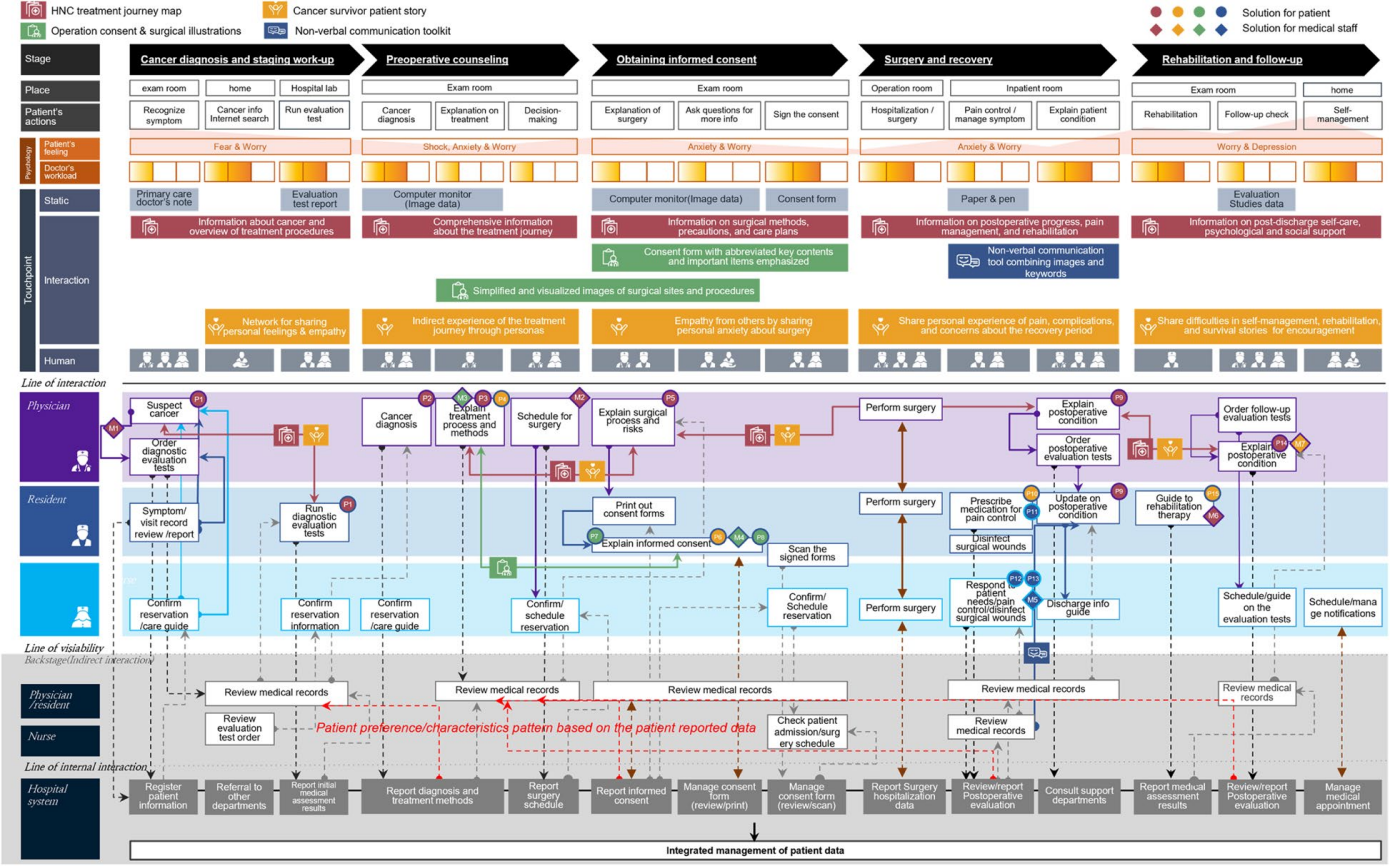
Improved HNC communication system by applying platform (To-be SB). (P: solution for patients corresponding to F, M: solution for medical staff corresponding to F’)

Figure 4 shows the integration of the proposed HNC communication platform with the current treatment system, presenting the integrative HNC communication system as a To-be SB. The proposed platform accumulated physical, psychological, and behavioral data collected from patients, while providing stage-specific solutions to meet their needs related to acquiring comprehensive information about the treatment journey, providing emotional support, delivering easily understandable information, and offering non-verbal communication methods. The accumulated data categorized by patient type within the system were analyzed to identify various patterns related to needs, preferences, and tendencies. This analysis provided insights for the medical staff, enabling them to efficiently formulate treatment plans and adopt patient-friendly approaches.

Considering that most HNC patients are older people with limited digital skills, we created solutions as simple as possible. To ensure patient access to the proposed platform, we took the measures such as information structuring and visualization to increase the readability, visual contrast and large fonts use for visibility, provision of information and alerts/notifications through SNS and text messages, compatible options between various electronic devices, and foldable leaflets and booklets to enable offline access. The notifications and alerts for patients are managed by sending automatic information messages to the contact information (mobile phone, email) that the patient initially registered. Medical staff will receive automatic notifications on the EHR system regarding the requirements entered by the patient during the hospitalization.

## Discussion

This study proposed a communication platform to facilitate effective information delivery and doctor–patient relationship improvement for HNC patients. To reflect a user-centered perspective, we conducted user research targeting patients, caregivers, and medical staff to identify service FPs for each journey stage. We found 22 service FPs (15 from patients and 7 from medical staff): 9 related to *comprehensive information delivery and easy access to care information*, 5 related to *emotional support to relieve negative feelings*, 4 related to *information visualization to understand the contents better*, and 4 related to *non-verbal communication tools to support active and prompt needs expression*.

Based on these four core needs, an HNC communication platform consisting of four solutions was developed: 1) *HNC treatment journey map*, 2) *Cancer survivor stories*, 3) *Operation consent form redesign with surgical illustrations*, and 4) *Non-verbal communication toolkit*. We conducted two focus groups with the HNC medical staff to validate the developed platform. We submitted our work to prestigious international design awards and received recognition through awards from Red Dot (2022) and iF (2023), affirming the value of the platform.

The effectiveness of the proposed platform can be confirmed in the following aspects.

First, based on a five-phased treatment journey, comprehensive information on the necessity for diagnostic tests, the content and flow of the entire treatment process, precautions before surgery, care plan after surgery, and rehabilitation management is provided to HNC patients. This enables easy access to treatment information for HNC patients, understanding and empathy for emotional changes, timely decision-making based on the reliable information directly provided by the medical staff, and systematic and efficient delivery of information in the form of a stage-by-stage structured map. This effect is consistent with Arnold et al. [46], who argued that the platform plays a positive role in addressing the unmet needs of cancer patients by providing reliable and easily understandable information while enhancing patient engagement.

Second, in survivor stories, HNC patients empathize with similar experiences to themselves through persona and cancer survivor storyboards, understand the patient's typical physical symptoms, emotional states, and procedures at each treatment stage, and easily check any questions they have through the information provided in the form of question and answer (Q&A). These results have the effect of increasing the patient's understanding and empathy for treatment information, forming a positive relationship between the patient and the medical staff, and promoting a positive commitment to treatment. These effects prove the positive role and effectiveness of emotional support of the proposed communication platform by actively reflecting the emotional support factors mentioned by Savioni et al. [47] such as the empathy for the patient’s negative emotions, good network connectivity among patients, and high interaction between doctors, patients and caregivers. Third, the operation consent form redesign with surgical illustrations included different color use according to the information hierarchy, medical terminology explanations in lay terms, creation of visual materials to easily explain treatment methods and surgical sites and highlight of the key points in bold and large fonts with increased line spacing. These results have the effect of enhancing HNC patients' comprehension of treatment and increasing treatment compliance by arousing positive attitude in treatment. In addition, the patient's understanding of the medical staff's explanation increases, which has the effect of improving trust and speeding up decision-making. This effect is evident in the results by suggesting specific solutions to practice the importance of communication between healthcare professionals and patients for optimal pain management as stressed by Phillips et al. [26].

Fourth, the non-verbal communication tool provides a means for patients to communicate with medical staff in the form of a booklet and app. The tool consists of five categories based on the patient’s requirements during the hospitalization combining simple language, images and symbols that reflect emotions. These results support the patient's active expression of their needs and opinions, allowing medical staff to quickly fulfill patient needs, thereby increasing patient satisfaction. These effects are considered in the same context as effective tracking patient behaviors and integrated treatment pathways establishment mentioned by Kouroubali et al. [9] as effects that can be achieved through a digital platform and support the effectiveness of the results of this study.

Improving work processes and sharing patient information among service providers can increase treatment adherence and alleviate staff workload, leading to better clinical experiences and health outcomes from the medical staff’s perspective. Doctors who convey technical terms and complex information to patients by identifying their interests and preferences increase their workload, as noted by several studies [48–52]. The proposed solution in this study automatically collects patient data, analyzes patient patterns, identifies trends, and presents primary data for clinical decision-making and patient response strategies in the early stages of treatment, thereby leading to an improvement in the HNC treatment process and enhancing patient satisfaction. As the number of users and data volume increases, the analysis results become more sophisticated and allow for classification of various patient types. The classified patient data provide the ground for setting the patient-customized treatment plan, patient preference, attitudinal approach, and judgement on when and how to utilize the solution. Hsiao et al.’s [5] perspective on the potential for enhancing healthcare service quality through user-friendly data collection tools and the presentation of integrated patient data into the workflow of medical staff supported the rationale behind our development of a communication platform. This platform integrates and analyzes the physical, emotional, and behavioral data of patients, and presents the results to the healthcare team. The trend in Health IT has increasingly emphasized a human-centered design approach. Carayon and Hoonakker [53] underscored the importance of teamwork among medical staff during a patient’s treatment journey. This further supports the practical value of a communication platform in which effective interaction and data-sharing occur among contact employees involved in the treatment journey.

However, a limitation of our study is that it focused on improving communication services, specifically for HNC patients. Furthermore, solutions for service enhancement are provided, concentrating on the three most common types of patients with HNC who often face communication challenges. Therefore, future studies should include a broader range of patient types and cases. The identified service FPs on effective information delivery and improvement in the doctor–patient relationship were gathered from a small number of patients and medical staff. Although the sample size is sufficient to ensure confidence in the research results, a significantly larger amount of user data collection and analysis is necessary for the proposed communication platform to be more sophisticated and to provide personalized services tailored to a larger patient population.

In addition to these limitations, future challenges that the current research needs to address include issues related to ensuring the security and privacy of the collected patient data, providing notifications and alerts to ensure patients are receiving the necessary information at the right time, and automating the data processing pipeline from collection and analysis to pattern interpretation and recommendations. Although the structure of the communication platform is visually represented through the SB and technical mechanisms such as data security and alert/notification were briefly introduced, there are still technical challenges to be overcome before it can be implemented. This study developed the communication platform targeting one of South Korea's representative hospitals. However, the size and applied systems of each hospital can vary, and depending on the culture, patients and medical staff may have different preferences for the communication methods and the appearance of the solution. Additionally, in order for this platform to be actively utilized in hospitals, shared decision-making, including recognition of the need for the platform and approval for the platform employment, must be made among stakeholders such as hospital administrators and service providers. The main focus of this study was the planning and designing a service-centric platform for information provision and relationship enhancement, so the detailed technical tasks required for implementation and operation remain subjects for future research.

## Conclusion

This study developed a communication platform to improve the quality of healthcare services for HNC patients, with a focus on information delivery and doctor–patient relationship improvement. To facilitate the practical development of the platform, service FPs were collected from both HNC patients and healthcare professionals. Based on this, specific solutions that were beneficial to both groups were presented. The significance of this study lies not only in proposing solutions through the developed platform but also in integrating it with the current treatment system. The SB illustrates the tangible outcomes of solution provision and the interactions between service providers, offering a comprehensive view of the proposed solutions. Furthermore, the significance of this study can be found in the comprehensive consideration of four aspects—informational, emotional, cognitive, and interactive—in structuring the platform, with a focus on aspects that contribute to service improvement.

Recently, many healthcare studies presented comprehensive views of recent advancements in healthcare service through the integration of advanced computational methods, showcasing novel approaches for imaging and diagnosis [54–56]. In terms of improving medical services through the combination of ICT, this study explores the significant contributions of HNC communication in providing effective information delivery and improving of doctor-patient relationship by integrating HNC communication platform into the EHR system. Additionally, the paper highlights the innovative use of service design techniques in medical domain, illustrating a holistic approach towards solving contemporary challenges in medical informatics and decision-making research topic.

A final contribution of this study lies in the development of a tailored platform for HNC patients with specific needs. It also applies design methodologies, such as co-creation design workshops and SBs, presenting flexible outcomes from a convergent perspective of medicine and service design.

The communication platform developed in this study proposes practical solutions to fulfill the four core needs of HNC patients: comprehensive treatment information provision, emotional support, information delivery in an easily understandable manner, and support for non-verbal communication. To provide comprehensive treatment information, we developed a map based on the five stages of the treatment journey, providing an overview of the entire journey and detailed information for each stage. To provide emotional support, we created HNC personas for virtual patients, allowing them to obtain various types of information and psychological empathy. To enhance the understanding of the information, we visually structured the operation consent form and incorporated surgical illustrations as explanations. Finally, for non-verbal communication support, we designed a highly usable booklet by developing an AAC system based on the needs frequently expressed by HNC patients.

In addition to these practical aspects, unresolved challenges that need to be specified for implementation such as patient data security, privacy, automation processing, and alerts remain. Future research should focus on specific approaches to address these unresolved technological issues. We anticipate the activation of a movement where patient data for improving communication for patients is collected in various hospital settings using the developed platform. Through the application of the proposed solutions, we expect a continuous enhancement in the quality of healthcare services.

## Data Availability

The datasets used and/or analyzed during the current study are available from the corresponding author upon reasonable request.

## Abbreviations

AAC: Augmentative and alternative communication
Apps: Applications
EHR: Electronic health records
FP: Failure point
HMW: How might we
HNC: Head and neck cancer
ICT: Information and communication technology
IT: Information technology
PE: Patient experience
SB: Service blueprint

## Footnotes

1 The communication platform that won the Design Award can be found at the following links: https://www.red-dot.org/de/project/augmentative-and-alternative-communication-toolkit-for-head-and-neck-cancer-patients-60983; https://ifdesign.com/en/winner-ranking/project/hnc-patient-centered-communication-platform/553339.
